# Ginsenoside Rb1 reduces oxidative/carbonyl stress damage and ameliorates inflammation in the lung of streptozotocin-induced diabetic rats

**DOI:** 10.1080/13880209.2022.2140168

**Published:** 2022-11-11

**Authors:** Hao Su, Cheng-Ju Tian, Ying Wang, Jiaojiao Shi, Xiaoxiao Chen, Zhong Zhen, Yu Bai, Lan Deng, Chunpeng Feng, Zhuang Ma, Jinfeng Liu

**Affiliations:** a Guang’anmen Hospital, China Academy of Chinese Medical Sciences, Beijing, PR China; b College of Physical Education and Sports Rehabilitation, Jinzhou Medical University, Jinzhou, PR China

**Keywords:** Diabetic lung, proinflammatory cytokines, apoptosis, lung injury

## Abstract

**Context:**

Ginsenoside Rb1 (Rb1) is a biologically active component of ginseng [*Panax ginseng* C.A. Meyer (Araliaceae)].

**Objective:**

This study determined the underlying mechanisms of Rb1 treatment that acted on diabetes-injured lungs in diabetic rats.

**Materials and methods:**

Streptozotocin (STZ)-induced diabetic rat model was used. Male Sprague-Dawley (SD) rats were divided into four groups (*n* = 10): control, Rb1 (20 mg/kg), insulin (15 U/kg to attain the euglycaemic state) and diabetic (untreated). After treatment for six weeks, oxidative stress assay; histological and ultrastructure analyses; TNF-α, TGF-β, IL-1 and IL-6 protein expression analyses; and the detection of apoptosis were performed.

**Results:**

There was decreased activity of SOD (3.53-fold), CAT (2.55-fold) and GSH (1.63-fold) and increased levels of NO (4.47-fold) and MDA (3.86-fold) in the diabetic group from control. Rb1 treatment increased SOD (2.4-fold), CAT (1.9-fold) and GSH (1.29-fold) and decreased the levels of NO (1.76-fold) and MDA (1.51-fold) as compared with diabetic rats. The expression of IL-6 (5.13-fold), IL-1α (2.35-fold), TNF-α (2.35-fold) and TGF-β (2.39-fold) was increased in diabetic rats from control. IL-6 (2.43-fold), IL-1α (2.27-fold), TNF-α (1.68-fold) and TGF-β (2.3-fold) were decreased in the Rb1 treatment group. Diabetes increased the apoptosis rate (2.23-fold vs. control), and Rb1 treatment decreased the apoptosis rate (1.73-fold vs. the diabetic rats). Rb1 and insulin ameliorated lung tissue injury.

**Discussion and conclusions:**

These findings indicate that Rb1 could be useful for mitigating oxidative damage and inflammatory infiltration in the diabetic lung.

## Introduction

It is known that diabetes affects various organ systems, such as the kidneys, retinae, nerves and cardiovascular system. Pulmonary function in diabetes has become a subject of interest because the association between diabetes and impaired lung function has been frequently observed. Various respiratory disorders have been described in patients with either type 1 and type 2 diabetes (Pitocco et al. 2012). Over the past two decades, clinical trials have shown clear decrements in lung function in patients with diabetes, and many reports have suggested plausible pathophysiological mechanisms (Hansen et al. 1989; Yamane et al. 2013; Rogliani et al. 2015; Sonoda et al. 2018). The most consistent pulmonary function abnormalities found in diabetics decreased lung volume and lung elasticity (Almeida et al. 2016). However, the potential molecular and cellular mechanisms responsible for diabetic lung have not yet been fully elucidated.

Excessive hyperglycaemia increases the imbalance between oxidative and antioxidative statuses within cells and tissues, which leads to an increase in reactive carbonyl species (RCS) and reactive oxygen species (ROS) (Tian and Zhen 2019). Oxidative stress and carbonyl stress are mechanisms responsible for the induction of pulmonary distress and lung dysfunction. Recent studies have demonstrated that the activity of superoxide dismutase (SOD), catalase (CAT) and glutathione peroxidase (GPx) was decreased, and malondialdehyde (MDA) and nitric oxide (NO) were significantly increased in lung injury (Samarghandian et al. 2014; Sharma et al. 2020). Emerging evidence suggests that antioxidants could be a possible treatment strategy for diabetes mellitus in male rats (Al-Salmi and Hamza 2021; El-Megharbel et al. 2022). These complicated mechanisms that govern the release of ROS and the accumulation of RCS in diabetic lung tissue damage are poorly understood.

Tumour necrosis factor alpha (TNF-α), transforming growth factor-β (TGF-β), interleukin-1 (IL-1) and interleukin-6 (IL-6) are well-known biomarkers of proinflammatory cytokines, which have also been found to be increased in respiratory disease. TNF-α is a multifunctional inflammatory cytokine that stimulates the release of IL-1β and IL-6 (Cuellar-Nunez et al. 2021). TNF-α may increase the expression of manganese SOD (Pogrebniak et al. 1991). Numerous studies report that IL-1, IL-6, TGF-β, TNF-α and ROS are mediators of deregulated inflammation and are implicated in respiratory disorder pathogenesis in lung injury models (Teixeira et al. 2008; Kilic et al. 2014; Khazri et al. 2016; Zhou et al. 2019; Karamalakova et al. 2022). In respiratory disease, mast cell degranulation increases IL-6 and TNF-α levels and immune cell numbers, which causes inflammation and impaired immune cell recruitment (Yang et al. 2019). Those investigations suggest that TNF-α, IL-6, ROS and RCS impart the lung structure observed in diabetic lungs.

Currently, there are relatively few pharmaceutical treatment options for diabetes-associated lung dysfunction, and those that are used to induce noteworthy side effects. As an alternative, treatments via traditional Chinese medicine (TCM) offer a variety of active ingredients that act upon multiple pathways and molecular targets. Ginseng [*Panax ginseng* C.A. Meyer (Araliaceae)] is a TCM that has been used in China for thousands of years. Ginsenosides are an important class of substances with biological activities found in ginseng, and the known ginsenosides are Rb1, Rg3, Re and Rg1. Rb1 is a biologically active component of ginseng that is responsible for its pharmacological properties (Bradford 1976; Tian et al. 2011; Bai et al. 2018; Dong et al. 2019; Fan et al. 2020). Rb1 (molecular formula: C_54_H_92_O_23_, MW: 1109.29 g/mol) is derived from the leaves, stems and roots of ginseng, and it exhibits a broad spectrum of pharmacological properties that prevent immune injury via alleviating oxidative stress and apoptosis (Rajput et al. 2021; Zhang et al. 2022). Numerous studies have demonstrated that Rb1 inhibits calcium entry into cells and affects the Nrf2 signalling pathway in human diseases (Wang et al. 2015; Ashrafizadeh et al. 2021). However, the underlying mechanisms conferring these efficacies remain poorly understood. Therefore, the present study used a diabetic rat model to investigate the mechanisms used by Rb1 in scavenging ROS and RCS, regulating TGF-β, TNF-α, IL-1 and IL-6, and ameliorating damaged lung tissue structure.

## Materials and methods

### Chemicals and drugs

**Figure 1.**
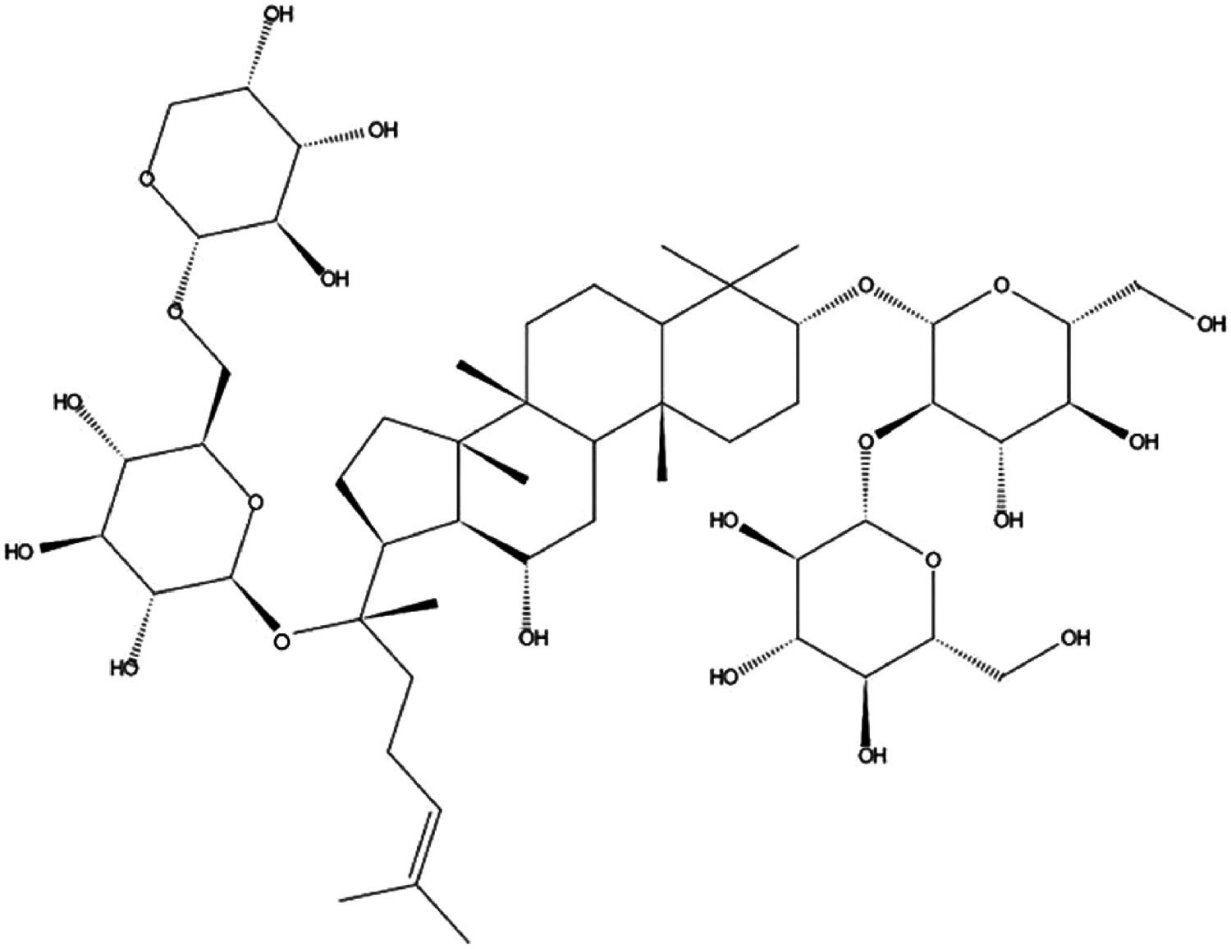
Types of ginsenosides Rb1.

SOD, CAT, GSH, NO and MDA assay kits were purchased from Jiancheng Biotech (Nanjing, China). Rb1 was purchased from Shanghai Yuanye Biotechnology Co., Ltd. (Shanghai, China) (batch number: p27S10U98131). The molecular construction of Rb1 is shown in Figure 1. The standard reagents and buffers used were of the highest grade available and were purchased from Sigma-Aldrich (St. Louis, MO).

### STZ-induced diabetic rat model and ginsenoside Rb1 treatment

In these experiments, 40 male Sprague-Dawley (SD) rats (170–180 g) were used. The SD rats were purchased from the Beijing Vital River Laboratory Animal Technology Co., Ltd. (Beijing, China). The rats were randomly assigned to a control group (*n* = 10) with normal feeding. Then, 30 rats were given a single intraperitoneal injection (0.25 mL) with freshly prepared streptozotocin (STZ) in a 2% solution of cold 0.1 M citrate buffer, pH 4.5 (45 mg/kg), to induce our rat model of diabetes as described earlier (Tian et al. 2011). The tail clipping method was used to measure the blood glucose of each rat a week later, and a blood glucose value >16.7 mmol/L was considered to be indicative of successful replication of the diabetic rat model. Two weeks after STZ injection, diabetic rats were randomly divided into three groups. One group (diabetic, *n* = 10) remained untreated. A second group (insulin, *n* = 10) was treated with insulin (15 U/kg) after 2 weeks of diabetes to attain euglycaemia. The third group (Rb1, *n* = 10) received intragastrically administered Rb1 (20 mg/kg of Rb1, Dong et al. 2019) after 4 weeks of diabetes. Animal use for the study was approved by the Institutional Animal Care and Use Committee of Jinzhou Medical University (case number JMU/EC/01/2018).

### Preparation of lung tissue

Eight weeks after initiating treatment, all rats were killed by exsanguination under isoflurane anaesthesia. After the thoracic cavities were opened in the midline, the lungs were exposed, detached and prepared for ROS/RCS, Western blot, and histochemistry staining studies.

### Lipid peroxidation and antioxidant defence system assays

Lung tissue was homogenized with KCl in a 1:10 ratio. The homogenate was centrifuged (9000×*g*, 30 min), and the supernatant was used for measurement of SOD, CAT, GSH, NO and MDA with commercially available assay kits (SOD, A001-3; CAT, A007-1-1; GSH, A006-2-1; NO, A012-1; and MDA, A003-1; Jiancheng Biotech, Nanjing, China) according to the manufacturer’s protocols. The total protein in each sample was measured according to the Bradford protein assay (Tian et al. 2011).

### Histochemistry staining

Histochemistry staining of lung tissues was performed as previously described (Temann et al. 1998). Briefly, the left lung was dehydrated, cleared, embedded in paraffin wax, and then was cut transversely into 7.0 µm-thick serial sections. Staining with haematoxylin–eosin (HE) and toluidine blue was performed, following the manufacturer’s instructions. The sections were finally observed and imaged under a light microscope.

### Transmission electron microscopy (TEM)

TEM was performed as previously described (Delgado-Buenrostro et al. 2013). Small specimens of lung tissue for each group of rats were collected and washed three times in PBS (10 min each). The tissues were fixed in 1% osmic acid in 0.1 M Na cacodylate buffer for 2 h at room temperature and soaked in epoxy resin for 2 h before embedding. The resin-embedded tissues were placed in an incubator with a thermostat for 24 h polymerization. The tissue blocks were serially sectioned with a thickness of 1 µm using an ultra-thin microtome. The ultrathin sections were stained with uranyl acetate and lead citrate, and then rinsed three times. The ultrastructure of the lung tissue for each group was observed using a JEOL 100 II transmission electron microscope (Boston, MA).

### Terminal deoxynucleotidyl transferase dUTP nick-end labelling (TUNEL) assay

The TUNEL assay was performed as previously described (Zhang et al. 2017). Briefly, apoptotic cells in the lung tissue sections were detected using a TUNEL kit (KeyGen, Nanjing, China) according to the recommendations of the manufacturer. Lung tissue samples were dissected from euthanized mice and stained using the TUNEL kit (KeyGen, Nanjing, China). The stained samples were independently observed under a Nikon E800 microscope by two pathologists (Tokyo, Japan). ImageJ software (Bethesda, MD) was used to count the numbers of 4′,6-diamidino-2-phenylindole (DAPI)-positive and apoptotic cells in six high-power microscope fields. Apoptotic cells were normalized by the total number of DAPI-positive cells.

### Western blotting analysis

Western blot analysis of TNF-α, TGF-β, IL-1α and IL-6 was performed as previously described (Liu et al. 2021). Briefly, lung tissue was lysed and mixed with immunoprecipitation lysis buffer. After complete lysis, the mixture was centrifuged at 13,000 rpm for 15 min to collect the supernatant, and the total protein was quantified via the bicinchoninic acid (BCA) assay. Western blot was performed using the following antibodies: anti-TNF-α 1:1000 (ab6671, Abcam, Waltham, MA), anti-TGF-β 1:1000 (ab92486, Abcam, Waltham, MA), anti-IL-1α 1:1000 (ab239584, Abcam, Waltham, MA) and anti-IL-6 1:1000 (ab233706, Abcam, Waltham, MA). The protein membrane was subjected to colour development via a chemiluminescent reagent, followed by film exposure and film scanning to measure the amount of relative protein. The Western blots were quantified using ImageJ (Bethesda, MD).

### Statistical analysis

Statistical analysis was performed using analysis of variance (ANOVA) employing SPSS 22.0 software (IBM SPSS Inc., Chicago, IL). The data were analysed by one-way ANOVA, followed by Bonferroni’s multiple comparison test. The data are presented as the mean ± SEM. In all cases, the level of statistical significance was set at *p* < 0.05.

## Results

### Rb1 affects the levels of SOD, CAT, NO, GSH and MDA in diabetic lung tissue

**Figure 2.**
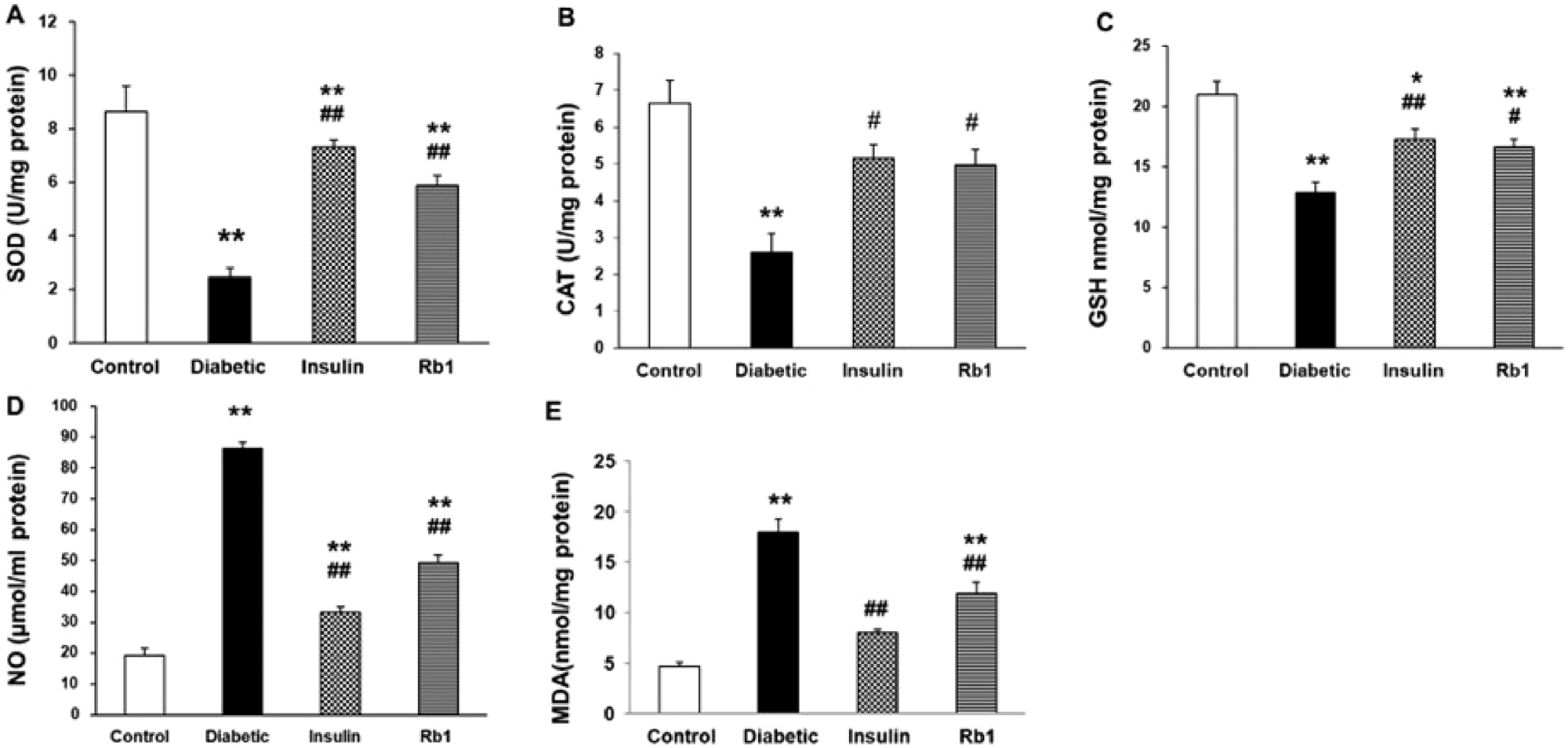
SOD, CAT and GPx activity and MDA level in the heart tissue. (A) SOD, (B) CAT and (C) GSH activity, and (D) NO level and (E) MDA level in the diabetic lung tissue of control, diabetic, insulin and Rb1 rats. The bars denote the mean ± SEM. *p < 0.05 and **p < 0.01 in the control rats; ^#^*p* < 0.05 in the diabetic rats; ^##^*p* < 0.01 in the diabetic rats.

The activities of SOD, CAT, NO, GSH, and the level of MDA in lung tissue are shown in Figure 2. SOD activity significantly decreased in the diabetic group (2.45 ± 0.35) and Rb1-treated diabetic (5.89 ± 0.38) group as compared with the control group (8.64 ± 0.97) (*p* < 0.01). SOD activity significantly increased in the insulin (7.32 ± 0.26) and Rb1 group as compared with the diabetic group (*p* < 0.01), as shown in Figure 2(A). CAT activity significantly decreased in the diabetic group (2.6 ± 0.5, *p* < 0.01) as compared with the control group (6.62 ± 0.64). The insulin group (5.13.79 ± 0.37) and the Rb1 group (4.94 ± 0.45) were not significantly different from the control group. There was a significant decrease in CAT activity in the insulin- and Rb1-treated diabetic group as compared with the diabetic group (*p* < 0.01 and *p* < 0.05, respectively), as shown in Figure 2(B). STZ injection produced a significant increase in NO compared with the control group (diabetic, 86.42 ± 2.11, control, 19.34 ± 2.17, *p* < 0.01). There was a significant decrease in NO in the lung homogenate of the insulin- (33.17 ± 1.82) and Rb1-treated diabetic groups (49.17 ± 2.59) compared with the diabetic group (*p* < 0.01), as shown in Figure 2(C). The level of MDA in the lung significantly increased in the diabetic group (17.93 ± 1.29, *p* < 0.01) and Rb1-treated diabetic group (11.87 ± 1.09) as compared with the control group (4.64 ± 0.36). The insulin (7.94 ± 0.38, *p* < 0.01) group was not significantly different from the control group. The level of MDA significantly decreased in the insulin- and Rb-treated diabetic groups as compared with the diabetic group (*p* < 0.01), as shown in Figure 2(D). The GSH activity in the lung homogenate significantly decreased in the diabetic group (12.88 ± 0.77, *p* < 0.01) and Rb1-treated diabetic group (16.59 ± 0.67, *p* < 0.01) as compared with the control group (20.97 ± 1.12). The GSH activity significantly increased in the insulin-treated diabetic group (17.28 ± 0.83, *p* < 0.01) and the Rb1-treated diabetic group (*p* < 0.05) as compared with the diabetic group, as shown in Figure 2(E).

### Rb1 normalized the morphological ultrastructure in diabetic lung tissue

**Figure 3.**
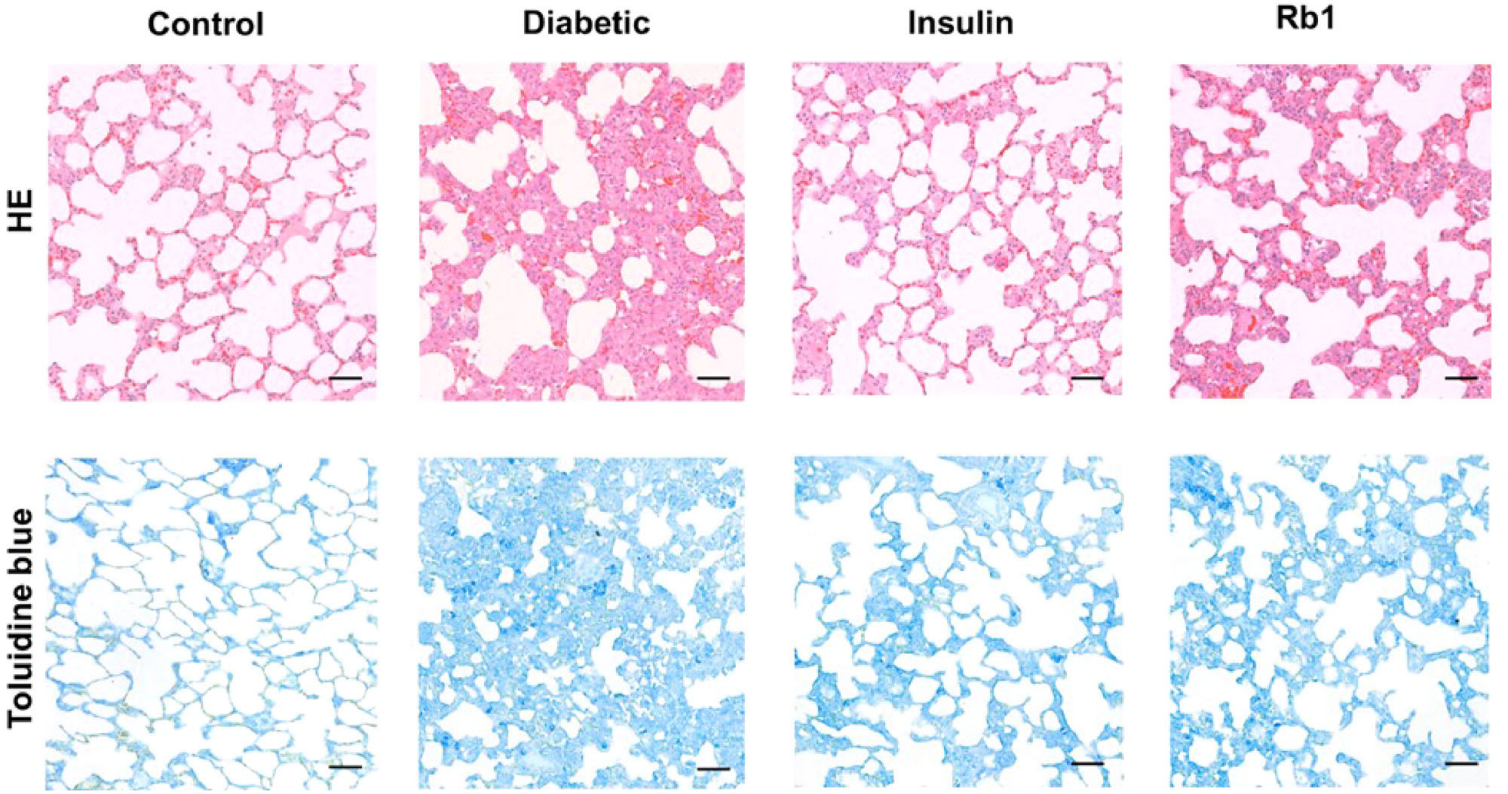
Morphological changes in lung tissue in diabetes mellitus. Control, diabetic, insulin and Rb1 treatment lung tissue of diabetic rats were stained with HE and toluidine blue. Bar = 50 µm.

As shown in Figure 3, H&E staining of the lung tissue revealed that normal control rats showed normal lung architecture with a normal bronchiole, thin alveolar septum, and alveoli lined mostly with squamous cells (type I pneumocyte) and cuboidal cells (type II pneumocyte). Compared with the control group, the diabetic group exhibited a loss of the normal lung architecture. Diabetic animals treated with insulin and Rb1 subsequently regained the normal architecture of the bronchiole and alveoli. Toluidine blue staining showed that there were more infected mast cells in diabetic rats. By contrast, there were less infected mast cells in insulin- and Rb1-treated diabetic rats.

**Figure 4.**
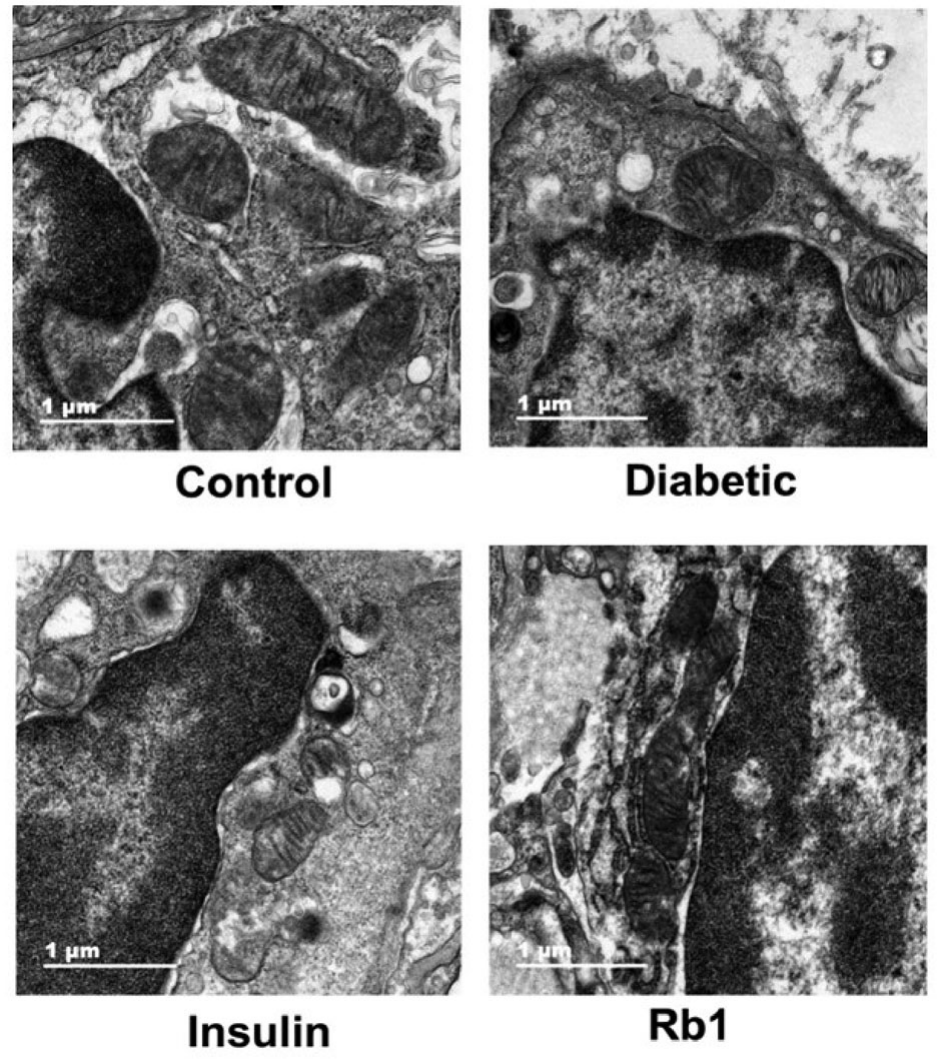
Representative results of mitochondria. Normal mitochondria from control rat lung tissue. Mitochondria were disordered and swollen in diabetic lung tissue, which was ameliorated by insulin and Rb1 treatment to varying degrees. Bar = 1 μm.

These data indicate that there was more severe inflammation in the lungs of diabetic rats, and thus there should be clear amelioration by Rb1 in the diabetic lung. The high magnification TEM image clearly shows visible mitochondria in control lung tissue. In diabetic lung tissue, many mitochondria were disordered and swollen, and some mitochondria were shrunken, with broken or missing cristae. In the insulin and Rb1 treatment groups, the mitochondria appeared more normal to varying degrees (Figure 4).

### Rb1 decreased apoptosis in diabetic lung tissue

**Figure 5.**
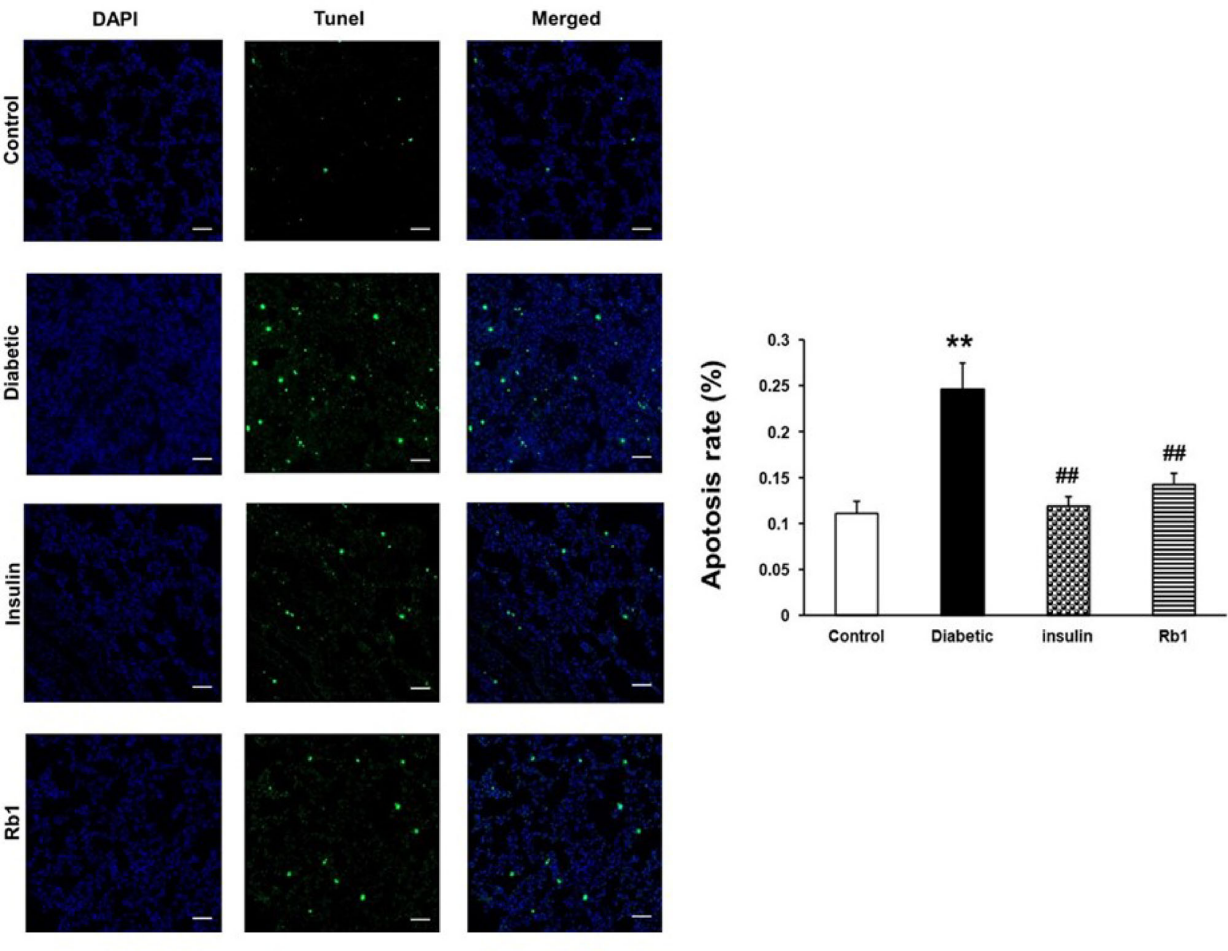
Rb1 decreases diabetic lung cell apoptosis. (A) Representative TUNEL staining of apoptotic cells in the control, diabetic, insulin and Rb1 groups. (B) The apoptosis rate in the four groups. The bars denote the mean ± SEM. **p < 0.01 in the control rats; ^##^*p* < 0.01 in the diabetic rats. Bar = 50 µm.

The apoptosis of diabetic lung cells was assessed by TUNEL staining. Representative TUNEL staining images from the four groups are shown in Figure 5(A). Quantitative analysis showed that the apoptosis rate was significantly increased in the lungs of diabetic rats compared with the control rats (*p* < 0.01). Insulin and Rb1 treatment significantly decreased the apoptosis rate compared with the diabetic rats (*p* < 0.01, Figure 5(B)). These results suggest that Rb1 inhibits lung cell apoptosis in a diabetic rat model.

### Rb1 decreased the expression of proinflammatory cytokines in diabetic lung tissue

**Figure 6.**
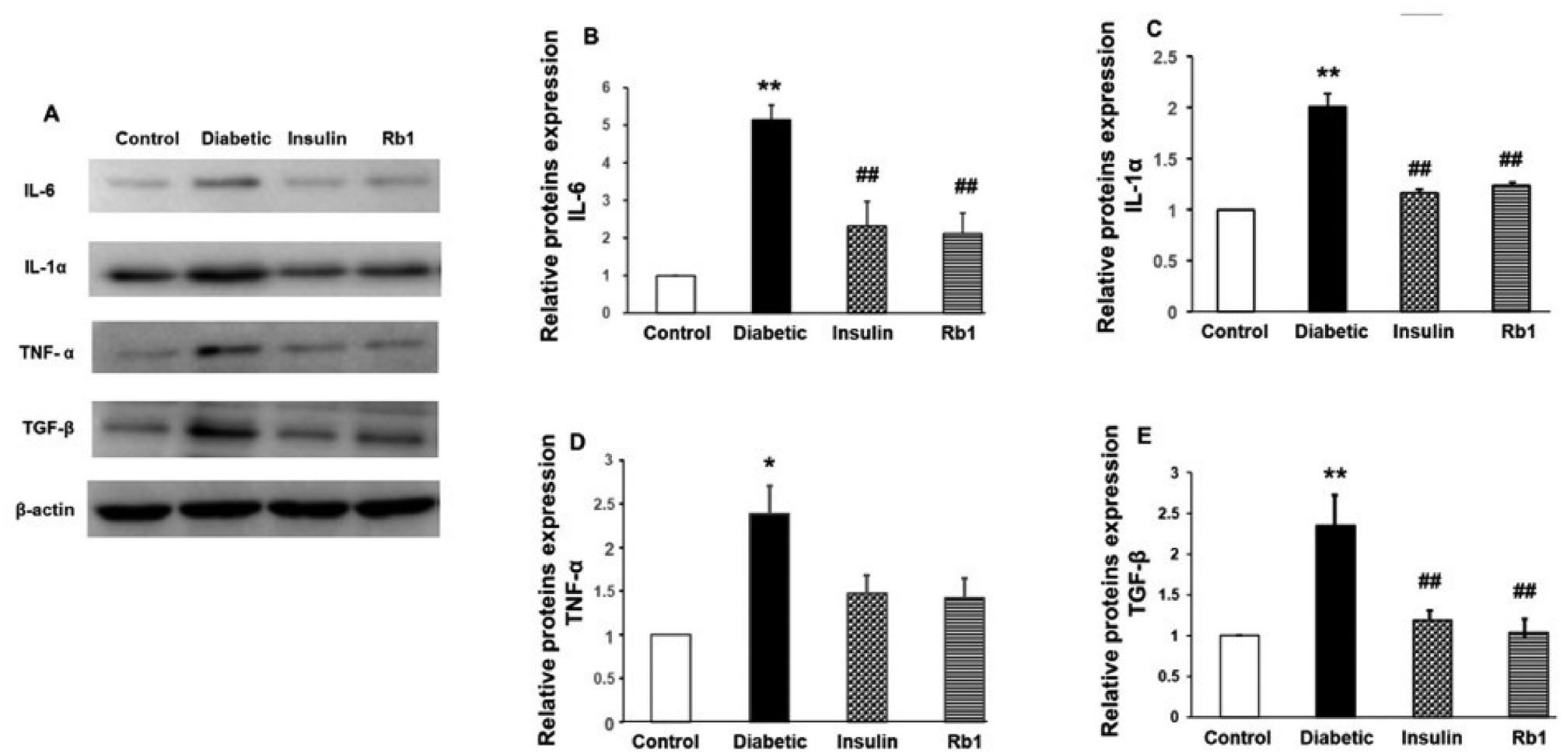
Decreased IL-1α, IL-6, TNF-α and TGF-β expression levels after treatment with Rb1. (A) Representative Western blots of IL-1α, IL-6, TNF-α, TGF-β and β-actin levels. (B) Graphs B–E show the mean ± SE of relative IL-1α, IL-6, TNF-α and TGF-β levels obtained from six separate preparations.

Representative expression of IL-6, IL-1α, TNF-α and TGF-β in the four groups is shown in Figure 6(A). The expression of IL-6 in the lungs of diabetic rats significantly increased by 5.13-fold (*p* < 0.01) compared with control rats. IL-6 expression in the insulin and Rb1 treatment groups was reduced by 2.22-fold (*p* < 0.01) and 2.43-fold (*p* < 0.01), respectively, compared with diabetic rats (Figure 6(B)). The expression of IL-1α in the lungs of diabetic rats significantly increased by 2.35-fold (*p* < 0.01) compared with the control rats. Insulin and Rb1 treatment resulted in significantly decreased IL-1α expression by 1.98-fold (*p* < 0.01) and 2.27-fold (*p* < 0.01), respectively, compared with diabetic rats (Figure 6(C)). The intensity of TNF-α expression in the lungs of diabetic rats was significantly increased by 2.39-fold (*p* < 0.05) compared with the control rats. TNF-α expression was reduced by 1.63-fold (*p* > 0.05) and 1.68-fold (*p* > 0.05) in the insulin and Rb1 treatment groups, respectively, compared with diabetic rats (Figure 6(D)). The expression of TGF-β in the lungs of diabetic rats significantly increased by 2-fold (*p* < 0.01) compared with control rats. TGF-β expression was decreased by 2.18- (*p* < 0.01) and 2.3-fold (*p* < 0.01) in the insulin and Rb1 treatment groups, respectively, compared with diabetic rats (Figure 6(E)). The increasing IL-6, IL-1α, TNF-α and TGF-β expression in the lungs of diabetic rats indicates that the expression of proinflammatory cytokines was upregulated in diabetes and was downregulated by insulin and Rb1 treatment.

## Discussion

Pulmonary complications in diabetes mellitus are greatly neglected because they are uncommon and not well recognized and may be life-threatening. Various respiratory disorders have been described in patients with either type 1 or type 2 diabetes (Goldman 2003; Pitocco et al. 2012). A recent study found that adults with either type I or type II diabetes are 8% more likely to have asthma, 22% more likely to have chronic obstructive pulmonary disease (COPD), and 54% more likely to have pulmonary fibrosis (Ehrlich et al. 2010). Although clinical trials have reported that lung and pulmonary complications exist in diabetes mellitus (El-Azeem et al. 2013; Mameli et al. 2021), the mechanism of diabetes-induced lung disorders has been poorly studied. Extensive experimental studies on the molecular mechanisms of diabetic lung are required to better understand the pulmonary risks in patients with DM and to improve medical management and the prevention of pulmonary complications in these patients.

The principal finding of the present study is that in the diabetic lung, there was increased oxidative/carbonyl stress, disrupted normal architecture of the lung, and upregulated expression of TNF-α and IL-6. During hyperglycaemia, normal metabolic processes are altered, with generation of oxidative stress and carbonyl stress (Tian and Zhen 2019). We found that the levels of SOD, CAT and GSH decreased and the concentration of MDA and NO increased in the diabetic lung. IL-6 and IL-1α mediate a wide range of inflammatory and immune responses and TNF-α and TGF-β confer proinflammatory effects on the lung. The Western blot showed high-level expression of IL-1α, IL-6, TNF-α and TGF-β in the diabetic lung. We also found abnormal lung architecture of the bronchioles and alveolar septum, including an increase in alveolar septal thickness and inflammatory exudate, in the diabetic lung.

Toluidine blue staining revealed that a greater number of infected mast cells were found in diabetic rats. TEM showed that the mitochondria were disordered and swollen, shrunken, and broken in diabetic lung tissue. The apoptosis of lung tissue increased in the diabetic rats. These data indicate morphofunctional alterations in the lung tissue of diabetic rats by mechanisms that involve increasing the oxidative stress, carbonyl stress and inflammatory infiltrate. This is consistent with other studies where TNF and IL-6 were necessary via targeting specific cytokines or cytokine-signalling pathways to reduce or ameliorate lung inflammation (Tutkun et al. 2019; Ma et al. 2020; Hirani et al. 2022). Mitochondria create most of the chemical energy needed to power the cell’s biochemical reactions. Dysfunction of mitochondria can generate excess ROS and RCS as a consequence of oxidative and carbonyl stress. ROS and RCS also result in the dysfunction of mitochondria, and mitochondria that are swollen and shrunken (Figure 5).

Parallel findings were also observed after insulin and Rb1 treatment. Insulin and Rb1 significantly increased the levels of SOD, CAT and GSH and decreased NO and MDA in the lung tissue of diabetic rats. The current study shows that insulin and Rb1 were effective in preventing the morphological alterations and decrease in mast cells typically observed in diabetic lung tissue. Ginsenosides are active ingredients in ginseng with immunomodulatory properties from cellular to organismal levels. In previous studies, Rb1 ameliorated the key inflammatory cytokines TNF-α and IL-6 in a cancer cachexia mouse model (Zhou et al. 2019; Lu et al. 2020). Rb1 also modulated lipopolysaccharide-induced proinflammatory cytokine production *in vitro* and *in vivo* (Smolinski and Pestka 2003; Cho et al. 2020). We confirmed which Rb1 significantly decreased the levels of IL-1α, IL-6, TNF-α and TGF-β in lung tissue.

These results show that Rb1 may have multiple mechanisms of action that normalize the mitochondrial structure and morphology and decrease apoptosis of the diabetic lung by reducing or scavenging the production of ROS and RCS and increasing the anti-inflammatory effect. The findings of this research are consistent with previous studies that designed therapy for diabetics with pulmonary dysfunction (Jiang et al. 2015; Qin et al. 2019; Refat et al. 2021).

## Conclusions

Our study presents evidence that indicates that increased oxidative/carbonyl stress leads to oxidative damage and inflammatory infiltration, which impairs the structure and function of the diabetic lung. Rb1 decreased oxidative/carbonyl stress and the expression of IL-1α, IL-6, TNF-α and TGF-β and normalized the morphology of the lung in the diabetic rats. These new data contribute to an increased understanding of physiologic/pathophysiologic mechanisms that occur in the diabetic lung.

## Data Availability

All data generated or analysed during this study are included in this published article.
